# Regional in vivo transit time measurements of aortic pulse wave velocity in mice with high-field CMR at 17.6 Tesla

**DOI:** 10.1186/1532-429X-12-72

**Published:** 2010-12-06

**Authors:** Marco Parczyk, Volker Herold, Gert Klug, Wolfgang R Bauer, Eberhard Rommel, Peter M Jakob

**Affiliations:** 1 Julius-Maximilians-Universität Würzburg, Lehrstuhl für Experimentelle Physik 5, Am Hubland, 97074 Würzburg, Germany; 2 Julius-Maximilians-Universität Würzburg, Medizinische Klinik und Poliklinik I, Oberdürrbacher Straße 6, 97080 Würzburg, Germany

## Abstract

**Background:**

Transgenic mouse models are increasingly used to study the pathophysiology of human cardiovascular diseases. The aortic pulse wave velocity (PWV) is an indirect measure for vascular stiffness and a marker for cardiovascular risk.

**Results:**

This study presents a cardiovascular magnetic resonance (CMR) transit time (TT) method that allows the determination of the PWV in the descending murine aorta by analyzing blood flow waveforms. Systolic flow pulses were recorded with a temporal resolution of 1 ms applying phase velocity encoding. In a first step, the CMR method was validated by pressure waveform measurements on a pulsatile elastic vessel phantom. In a second step, the CMR method was applied to measure PWVs in a group of five eight-month-old apolipoprotein E deficient (ApoE^(-/-)^) mice and an age matched group of four C57Bl/6J mice. The ApoE^(-/-) ^group had a higher mean PWV (PWV = 3.0 ± 0.6 m/s) than the C57Bl/6J group (PWV = 2.4 ± 0.4 m/s). The difference was statistically significant (p = 0.014).

**Conclusions:**

The findings of this study demonstrate that high field CMR is applicable to non-invasively determine and distinguish PWVs in the arterial system of healthy and diseased groups of mice.

## Background

Cardiovascular diseases are among the most common causes of death in industrialized countries. Risk factors include increased age, male sex, diabetes, hypertension, and lipoprotein abnormalities. The aorta provides at least 60 to 70% of systemic compliance [1]. Reduced elasticity and compliance of the aorta are etiologic in cardiovascular diseases such as atherosclerosis and therefore serve as early indicators of asymptomatic atherosclerotic lesions [2,3]. The velocity of pressure and flow pulses travelling down an elastic vessel termed the pulse wave velocity (PWV) increases with arterial stiffness [4]. The PWV is a direct measure of arterial stiffness [3] and serves as an independent predictor for cardiovascular risk and mortality [5-8] in many cases of CVD, including atherosclerosis [9]. In this study, a high field cardiovascular magnetic resonance (CMR) protocol using the transit time (TT) method [10] was developed and tested for its capability to distinguish groups of healthy and atherosclerotic mice by means of the PWVs in the descending aortas.

Originally, the transit time method has been used to determine the PWV in humans using several invasive methods [11,12] and non-invasive methods such as ultrasound [13,14] and CMR [15] before it was applied to smaller mammals such as mice. To this day new CMR methods have been refined by many workgroups [16,17] and they constitute a comprehensive technique for the characterization of morphologic and functional arterial systemic parameters in humans.

In the past, the need to determine morphologic and functional parameters of the murine arterial system arose. Genetically engineered phenotypes of mice that are deficient in apolipoprotein E (ApoE^(-/-) ^mice) spontaneously develop severe hyperlipidemia and atherosclerotic lesions at the arterial wall [18]. ApoE^(-/-) ^mice develop all stages of lesions observed during atherogenesis that resemble lesions found in humans [19]. Therefore and for its short generation times, the ApoE^(-/-) ^mouse model became an important model for human atherosclerosis [20].

Physical dimensions in mice are approximately 20 times smaller than in humans. With isoflurane anesthetized mice have high heart rates of 8 to 10 beats per second, whereas healthy humans at rest have heart rates of 0.8 to 1.7 beats per second. The necessities of very high spatial and temporal resolutions constitute the challenges for the determination of the PWV. In CMR of the corresponding animal model, the acquirable spatial resolution is limited by the signal-to-noise ratio (SNR). In the past, CMR systems did not provide a sufficient SNR to allow for accurate PWV measurements in mice. New high-field CMR systems facilitate a very high SNR and allow for examinations of morphologic and also functional parameters of the arterial system of mice [21,22].

Alternatively to CMR, ultrasound methods have been utilized to non-invasively measure PWVs in mice [23-25]. However, these ultrasound methods turned out to be highly observer dependent and in general were limited by acoustical windows in the thorax and by angular offsets in the alignment of the ultrasound transducer and the aorta [26]. The limited spatial resolution of ultrasound is a substantial impediment for morphological studies [27].

The primary objective of this study was to test the applicability of CMR and the TT method to assess the regional PWV of the descending murine aorta. The MR measurements were performed on a CMR system with a main magnetic field of 17.6 T. The accuracy and reproducibility of the CMR method were validated on a vessel phantom made of poly(vinyl alcohol) cryogel (PVA-C). In addition, it was tested whether the precision of the CMR method is sufficient to differentiate the mean PWVs of groups of atherosclerotic ApoE^(-/-) ^and healthy wild type mice.

## Methods

### The Transit Time Method

During each systole the left ventricle of the heart ejects one stroke volume of blood into the aortic root, hence, generating a pulse beat. The pulse beat that in fact is a local increase of blood pressure and flow velocity propagates down the aorta with a velocity that is named the pulse wave velocity (PWV).

**Figure 1:**
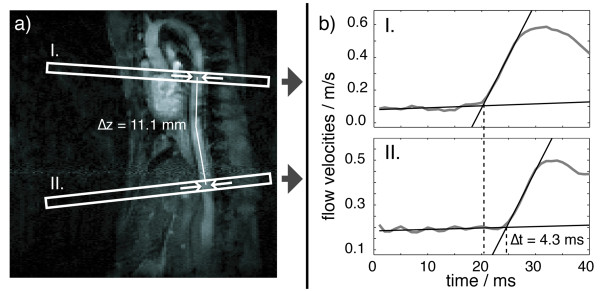
**In vivo PWV measurements in the descending murine aorta**. Figure 1a shows a reference scan of the murine aorta and the positioning of velocity measurement slices (I: thoracic aorta, II: abdominal aorta). Arrows indicate the regions of velocity measurements (two-point TT method). Figure 1b depicts representative flow velocity waveforms of an ApoE^(-/-) ^mouse and the regression lines that determine the pulse onset time. Only the late diastole and the early systole were recorded. This particular measurement yields a PWV = Δz/Δt = 2.6 m/s.

The principle to measure arterial PWV with the TT method is as follows. At least two measurement sites, I and II in Figure 1a, are to be selected on the aorta at a mutual distance, Δz. The transit time, Δt, which the pulse needs to travel from location I to II is to be measured (see Figure 1b). The PWV is calculated as PWV = Δz/Δt. This procedure is called the two-point TT method.

The generalization of the two-point TT method is the multi-point TT method [28-30]. It measures the transit times of the pulse waves in multiple locations along the path of wave propagation. The PWV is the constant of proportionality between the distances and the corresponding transit times.

**Figure 2:**
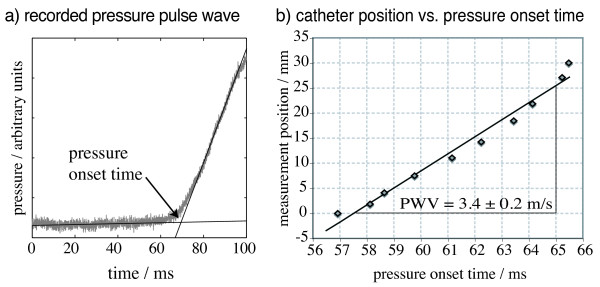
**Pressure wave measurements on the vessel phantom**. Figure 2a shows a representative time course of the pressure wave recoded on the vessel phantom (sample rate = 100 ms^-1^). Figure 2b illustrates the transit of the pressure wave. In order to depict the PWV as the slope of the linear regression line, the independent variable, i.e., the pulse onset time is exceptionally plotted along the abscissa. The diagram shows one of six series of measurements that were performed at different installation heights (here +2 mm) of the vessel phantom relative to the MR iso-center.

Figure 1b and 2a reveal that flow and pressure pulse curves show a gradual rather than an instantaneous increase. However, a distinctive feature, the foot of the pulse wave, is necessary to determine the transit time. The foot of the pulse wave is defined as the intersection point of a regression line fitted to the velocity or pressure values before the pulse and another line fitted to the early increasing values.

### Phantom Experiments

**Figure 3:**
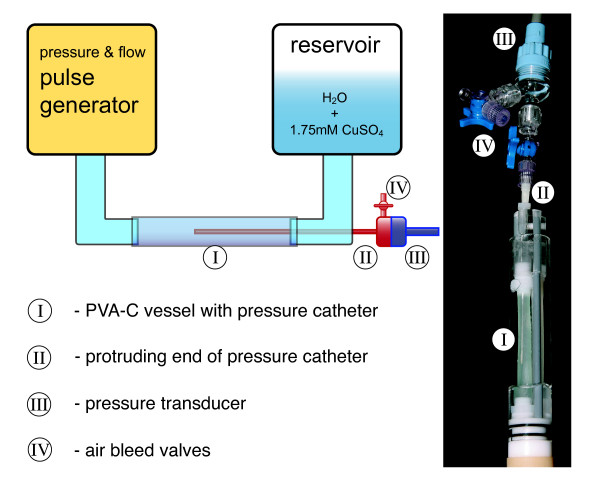
**Simplified scheme and photograph of the pulsatile elastic vessel phantom**. The phantom was used to investigate the accuracy and the precision of the developed MR TT method.

The accuracy of the MR method was tested on an elastic vessel phantom that was connected to a custom built flow and pressure pulse generator. The phantom allowed for the determination of PWVs by CMR measurements of flow waveforms and for reference measurements of pressure waveforms utilizing a pressure catheter that was inserted in the outlet end of the vessel (Figure 3). The PWVs obtained by the two different measurement methods were compared to each other in order to give evidence of the accuracy and precision of the MR method.

The phantom material had to have appropriate physical properties, e.g., mechanical strength, uniformity, long-term stability, and a modulus of elasticity resembling animal tissue. Poly(vinyl alcohol) cryogel (PVA-C), discovered independently by Peppas et al. and Nambu et al. [31,32], has high breaking strengths and physiologic elastic moduli ranging from 0.1 to 1.0 MPa [33] and was, therefore, used to build the phantom vessel. Aqueous poly(vinyl alcohol) solution (15% w/w) was injected into an acrylic mold and crosslinked to form the gel by repeated freezing and thawing. The elastic modulus of the gel depends amongst others on the number of the freeze-thaw cycles, on the concentration of the aqueous solution, and essentially on the thaw rate [33,34].

The vessel phantom had an inner diameter of 6 mm and a wall thickness of 0.25 mm. Its length was approximately 8 cm. The vessel was dimensioned according to Eq.[1], the Moens-Korteweg equation [35], to have a PWV in the physiological range of 2.0 to 6.5 m/s.

(1) $$PWV=\sqrt{\frac{Eh}{\rho d}}$$

E is Young's modulus of elasticity, h and d are the vessel wall thickness and the diameter, and ρ is the fluid density. The phantom was stored in a water bath to stabilize for at least 14 days prior to measurements because PVA-C is subject to an aging process that can affect its elasticity [34].

A homemade pressure and flow pulse generator was connected to the inlet end of the vessel phantom. A function generator (F34, Interstate Electronics Corporation, Anaheim, CA, USA) controlled the pulse generator. The function generator was set to generate pressure pulses every 1.8 s. Therewith, residual waves reflected at impedance mismatches inside the vessel phantom setup could decay completely between pulses. The function generator also triggered the data acquisitions of the MR system and of the setup used to record the pressure waves inside the vessel phantom.

The setup to record pressure waves comprised a pressure catheter, a signal amplifier, and a storage oscilloscope. The catheter was compounded of a blunted canula (TSK-Supra, Ebhardt-Söhne GmbH, Geislingen, Germany) and a Statham P23XL pressure transducer (Viggo-Spectramed, Inc., Oxnard, CA, USA). The signal amplifier was an MBS STAT ZAK (ZAK Psychologische und Physiologische Instrumente GmbH, Simbach, Germany). The storage oscilloscope was a TDS 3032 (Tektronix Inc., Beaverton, OR, USA).

The outlet end of the vessel was connected to a reservoir filled with an aqueous solution of 1.75 mM CuSO_4_. The height of the reservoir was adjusted in order that the vessel did not bloat or collapse. Two plastic rods supported the vessel on one side to prevent it from swinging in the transversal direction but allowed it to expand during the transit of the pulse.

The multi-point TT method was used to determine the PWV of the vessel phantom by pressure measurements. The measurement positions of the pressure catheter were determined at its protruding end with a calliper gauge. The pressure data were analyzed in MATLAB (The Mathworks, Inc., Natick, MA, USA) to determine the onset times of the pressure waves for each measurement position of the pressure catheter. The measurement positions were plotted versus the corresponding pressure pulse onset times. The slope of the linear regression yielded the PWV (see Figure 2b). The multi-point TT measurements were performed at six different installation heights of the vessel phantom that ranged from -13 mm to 12 mm from the MR iso-center. The PWV at the MR iso-center was determined by linear regression through the PWV and installation height values.

The MR system that was used for the phantom and in vivo measurements was a Bruker AVANCE 750 spectrometer (Bruker Biospin, Rheinstetten, Germany) with a vertical main magnetic field of 17.6 T and a bore size of 89 mm. The self-shielded gradient insert, a Bruker Micro 2.5, had a maximum gradient strength of 993 mT/m and an inner diameter of 40 mm. The homebuilt transversal electromagnetic mode radio frequency (RF) resonator had an accessible inner diameter of 25 mm.

**Figure 4:**
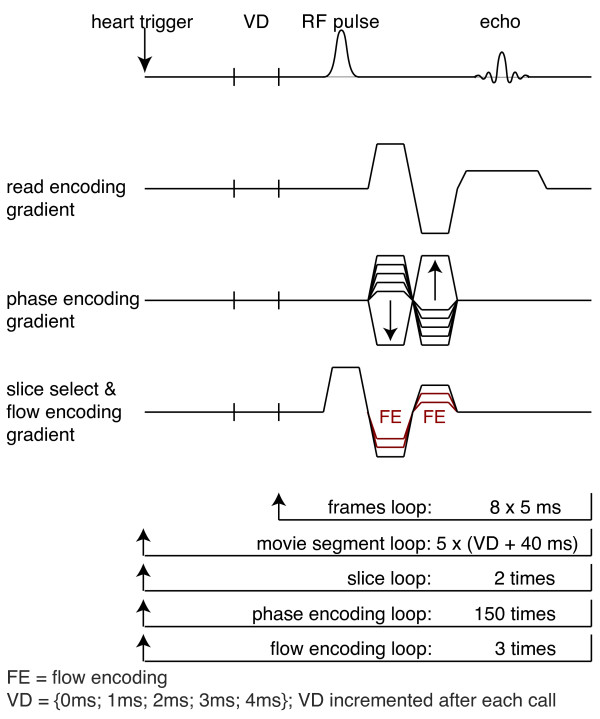
**Scheme of the flow encoding MR method used to measure the PWV in the descending murine aorta**. A fivefold repetition of the frames loop with interjacent incrementation of the variable delay produced an effective temporal resolution of 1000 time frames per second.

The MR sequence applied phase velocity encoding to record the time courses of the flow velocities. The sequence was based on a two-dimensional FLASH sequence with incorporated velocity compensating gradients in all three gradient directions (Figure 4). To encode through-slice flow velocities, bipolar velocity encoding gradients were superposed onto the velocity compensating gradients in the slice direction. Three flow encoding steps were applied to each scan. The velocity-encoding window of the MR sequence was set to ± 0.20 m/s to accommodate the maximal flow velocities. The flow encoding gradients required the maximal available gradient power.

Transit times of the pulse waves are in the range of a few milliseconds, therefore, a temporal resolution of 1 ms was necessary to be able to obtain the PWV from the time courses of arterial blood flow velocities. The intrinsic temporal resolution of the MR sequence, equivalent to its repetition time, was 5 ms. Hence the time courses of the flow velocities had to be sampled in an interleaved fashion. The sequence was initiated five times, every time with an additional delay of 1 ms between the trigger signal and the initiation of the sequence. The resulting five data streams were interleaved in the post processing to generate a recording of flow velocities with a temporal resolution of 1 ms. The detailed timing and loop structure of the MR sequence are visualized in Figure 4.

The spatial in-plane resolution was 147 × 147 μm^2 ^and the slice thickness was 1 mm. The field of view (FOV) was 22 × 22 mm^2^. The echo time was 1.6 ms. The Gauss RF excitation pulse had a length of 200 μs and an excitation angle of 20°. Signal averaging was not applied.

The two-point and multi-point TT methods were used to determine the PWV of the vessel phantom by MR measurements. A set of two-dimensional Fast Low Angle Shot (FLASH) experiments was leading the measurement protocol to localize the measurement positions on the vessel phantom. The MR imaging slices were positioned perpendicularly to the vessel at positions ±2.5 mm, ± 5.0 mm, ± 7.5 mm, and ± 10.0 mm from the magnets iso-center. A perpendicular slice orientation was crucial to avoid displacement artifacts caused by fluid flowing out of the imaging slice.

MR data were processed with a custom written routine using MATLAB. Velocity information was computed for pixels inside the vessel lumen by fitting a line to the phase values as a function of the first moments of the velocity encoding gradients (phase difference method). For every time frame the velocities were averaged over the luminal area.

64 pressure measurements were performed before 80 MR measurements. Differences in the mean PWV values obtained on the vessel phantom by the two measurement methods were tested by a two-sided t-test for unpaired data points for the hypothesis of equality against the alternative of differing values. The data points were not paired because many parameters, such as static pressure, temperature, and acoustic noise could not be held constant during the transition from pressure to MR measurements due to limited access and the strong magnetic field inside the magnet. The hypothesis was accepted for p-values ≥ 0.05. PWV values are given as mean ± standard error (SE).

### In Vivo Experiments

The proposed MR method was designed to utilize the two-point TT method in vivo in the descending murine aorta. Nine MR measurements were performed on a group of five female eight-month-old ApoE^(-/-) ^mice and eight measurements on a group of four age- and sex-matched C57Bl/6J mice. The ApoE^(-/-) ^mice were fed a western type diet (TD 88137, Harlan Laboratories, Inc., Indianapolis, IN, USA) 10 weeks prior to MR measurements. During the MR examinations, the mice were anesthetized with an isoflurane inhalation (1.5 - 2.0 Vol.%) in O_2 _(2 L/min) applied by means of a nose cone. Mice were placed vertically (head up) in the RF resonator. Due to the small diameters of the gradient insert and the RF resonator, their body temperature could be kept constant at 37°C by adjusting the temperature of the gradient insert temperature control unit.

A pressure sensitive pneumatic balloon (Graseby Medical Limited, Watford, United Kingdom) was placed between the inner RF resonator wall and thoraces of mice to detect cardiac trigger and respiratory gating signals. Outside of the gradient insert, a pressure transducer (24PCEFA6 D, Honeywell S&C, Golden Valley, MN, USA) transformed the pressure signal from the balloon into an electrical signal that was amplified and processed in real-time by a homebuilt unit. Thus, electrical interference between the trigger signal and gradient fields oscillating in the same frequency domain was avoided.

All experimental procedures were in accordance with institutional and internationally recognized guidelines and were approved by the Regierung von Unterfranken (Government of Lower Franconia, Germany). The reference number of the permit of the animal experiments is 55.2-2531.01-19/07.

Two imaging slices were positioned perpendicularly to the thoracic and the abdominal aorta (as illustrated in Figure 1a). Reasonable in vivo experiment times only allowed for the two-point TT method to be applied. Slice separations were maximized in order to minimize the errors in the measured PWVs. Again, a perpendicular slice orientation was of crucial importance. To localize the descending aorta, a set of two-dimensional FLASH experiments was leading the measurement protocol.

The velocity-encoding window was set to ± 1.66 m/s to accommodate the maximal blood flow velocities. The RF excitation pulse had an excitation angle of 40°. The mice had a heart period of approximately 115 ms. A time window of 40 ms was sufficient to sample the late diastole and early systole. All other parameters of the measurement protocol were set as in the measurements on the vessel phantom. The total image acquisition times ranged from 15 to 20 min.

MR data were processed using the MATLAB routine that was also used for the analysis of the data acquired on the vessel phantom. The curve progression in the velocity-time diagrams showed sharp bends between the sections before and during the pulse. This allowed for a semi automatic selection of the fit ranges that defined the onset times of the flow pulse. The fit range before the pulse was selected manually. It included data points on an approximate horizontal line. The beginning of the pulse fit region was set automatically as a series of three consecutive data points with velocity values at least two standard deviations above the extrapolated fit line of the section before the pulse. The end point was selected manually as the last data point on the first straight portion of the flow pulse section. The program AMIRA (Visage Imaging, Inc., San Diego, CA, USA) was applied to gauge the distance between the two measurement locations. Therefore, straight-line segments were drawn along the luminal midline in a longitudinal reference scan of the aorta.

Isoflurane causes respiratory depression and, therefore, anesthetized mice develop a gasping breathing pattern with a period of approximately 1 s to 1.5 s. The duration of each respiratory movement is approximately 0.4 s. The MR data were acquired in the intermediate movement-free time intervals. From the periodicity of previous respirations, the homebuilt heart-triggering/breath-gating unit anticipates the next phase of respiratory motion and stops the MR data acquisition before the respiratory motion sets in. Data acquisition is continued approximately 0.4 s after the detected onset of breathing motion. Occasionally, the breathing pattern was not precisely periodic. Then data was acquired during the respiratory movement and motional artifacts in the MR images were the result. Sporadic non-periodic respiration was observed during all measurements, but showed no adverse motional artifacts in most of the measurements. In some of the motional artifacts, the signal amplitude and phase information of extra-luminal pixels were shifted into the vessel lumen. Those pixels were excluded from the calculation of the flow velocities when the anatomical structures that those pixels belonged to could be identified visually.

Differences in the mean PWV values of the two animal groups were tested by a two sided t-test for the hypothesis of equality against the alternative of a higher value for the ApoE^(-/-) ^group. The hypothesis was rejected in favor of the alternative for p-values < 0.05. In this work all in vivo PWV values are given as mean ± standard deviation (SD).

## Results

### Phantom Experiments

On the vessel phantom, a reference PWV = 3.31 ± 0.18 m/s was obtained by the pressure catheter measurements. The multi-point MR measurements determined a PWV = 3.32 ± 0.18 m/s. The PWV values of the pressure and MR measurements are not different with statistical significance (p = 0.999). The parameters and results of the validation measurements are summarized in Table 1. The PWV values of the two-point MR measurements agree within their standard errors. A representative pressure wave in the vessel phantom and the multi-point TT method (explained in the method section) are shown in Figure 2.

**Table 1:** Parameters and results of the validation PWV measurements.

pressure TT measurements		
number of data points	measurement positions	PWV
64	multi-point	3.31 ± 0.18 m/s*

flow velocity TT method		
number of data points	measurement positions	PWV
80	multi-point	3.32 ± 0.18 m/s*
20	two-point at ± 10.0 mm	3.30 ± 0.20 m/s
20	two-point at ± 7.5 mm	3.39 ± 0.49 m/s
20	two-point at ± 5.0 mm	3.17 ± 0.29 m/s
20	two-point at ± 2.5 mm	3.44 ± 0.94 m/s

PWV values are given as mean ± standard error. *The mean values of the pressure and MR measurements do not show a statistically significant difference (p = 0.99).

### In Vivo Experiments

**Figure 5:**
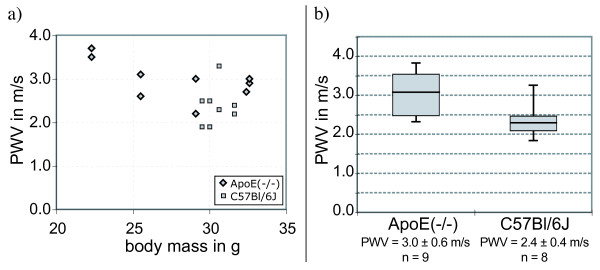
**PWV values in descending murine aortas**. Figure 5a shows the individually measured PWVs of ApoE^(-/-) ^and C57Bl/6J mice plotted versus the body masses. Vertically aligned datapoints belong to the same animal. Figure 5b shows a box-and-whiskers plot of the PWV values of the groups of eight-month-old female ApoE^(-/-) ^and C57Bl/6J mice. The whiskers indicate the minimum and maximum values in each animal group. The boxes indicate the lower and upper quartiles; the band near the middle of each box represents the median. The ApoE^(-/-) ^mice received a western type diet 10 weeks prior to the measurements. The PWV values are stated as mean ± standard deviation. The number of measurements on each group is indicated by **n**.

Figure 1 shows representative time courses of flow velocities, which were recorded on an ApoE^(-/-) ^mouse. The measured PWV values (Figure 5) ranged from 1.9 m/s in a C57Bl/6J mouse to 3.8 m/s in an ApoE^(-/-) ^mouse. The average values of each animal group are PWV = 3.0 ± 0.6 m/s for ApoE^(-/-) ^mice and PWV = 2.4 ± 0.4 m/s for C57Bl/6J mice. The mean value of the ApoE^(-/-) ^group was higher than that of the C57Bl/6J group with statistical significance (p = 0.014). The change of the heart periods of examined mice was smaller than 5 ms during experiments. The experiment parameters and the results for both animal groups are summarized in Table 2.

**Table 2:** Parameters and results of the in vivo MR PWV measurements.

parameter (mean ± standard deviation)	**ApoE **^**(-/-)**^**group**	C57Bl/6J group
age of animals (mo.)	8	8
number of animals	5	4
number of measurements	9	8
body mass (g)	28.4 ± 4.5	30.4 ±0.9
anesthetic		
isofluorane (%)	1.8 ± 0.2	1.7 ± 0.2
in O_2 _(l/min)	1.5 ± 0.0	1.8 ± 0.4
avg. heart period (ms)	109 ± 9	112 ± 4
change in heart period of single experiments (ms)	2.0 ± 1.6	2.2 ± 1.5
distance between measurement locations (mm)	12.2 ± 1.1	9.5 ± 1.4
transit time between measurement locations (mm)	4.1 ± 0.9	4.2 ± 1.1
PWV* (m/s)	3.0 ± 0.6	2.4 ± 0.4

*The mean PWV of the ApoE^(-/-) ^group is higher than the mean PWV of the C57Bl/6J group with statistical significance (p = 0.014).

## Discussion

A pulsatile elastic vessel phantom with a physiological PWV was developed. PWV values of the vessel phantom were investigated by MR and pressure measurements. The mean PWV determined by the MR multi-point TT method showed no statistically significant difference to the reference value. The developed MR TT method is accurate. The agreement of the MR two-point TT measurements at the different measurement positions on the vessel phantom indicates that the MR method delivers reproducible PWV values. These measurements also indicate that the separation of the imaging slices shall be maximized in order to reduce the uncertainty in the measured PWV. A statement about the in vivo precision of the MR method in a best-case scenario cannot be made from the results of the phantom measurements, because the standard deviation of the phantom measurements is higher than that of the in vivo measurements. Different error mechanisms, such as residual transversal vessel wall oscillations [36], water droplets forming on the outside of the phantom vessel wall, and a jitter in the pressure pulses reduced the precision of the phantom measurements.

The results of this study show that the PWV is measurable in vivo in the descending murine aorta using a two-point TT CMR method and an CMR system with a main magnetic field strength of 17.6 T. The measurements are made possible by the high SNR intrinsic to high-field CMR and the large filling factor of the used RF resonator. The interleaved acquisition scheme and the pneumatic triggering technique (explained in the methods section) provide the necessary temporal resolution. Heart periods are sufficiently stable throughout the experiments, due to reliable anaesthesia and body temperature control. Flow compensation and the short echo time of the MR sequence greatly reduce motional artifacts. Additionally, the short echo time alleviates susceptibility artifacts, which are usually caused by boundaries of tissues with differing magnetic susceptibilities in high-field CMR.

Wave reflections, mainly generated by the micro-vascular bed, i.e. the arterioles, will change the shape of the flow wave and will affect the estimation of PWVs [37,38]. It is critical for the determination of the PWV of the forward travelling pulse wave that only the reflection-free part of the wave is analyzed. Assuming a hypothetical PWV of 5 m/s and a distance of 20 mm between the abdominal measurement position and the microvascular bed, the reflected pulse wave needs 8 ms to return to the abdominal aorta. In this study, at most the early 7 ms of the flow velocity upstroke were used for the calculation of the PWV, therewith, wave reflections did not impose an impediment.

The PWV measured on the group of eight-month-old ApoE^(-/-) ^mice was 25% higher than the PWV in the age-matched wild-type group. The in vivo experiments showed, that the precision of the measurement method suffices to distinguish between the PWVs of different animal groups. The difference is statistically significant (p = 0.014). The PWV values measured in mice in this study are lower than values previously stated in literature but agree in general with most publications [25,39,40]. The deviations between the findings of this study and literature might be caused by systematic deviations inherent to the different methods of measurement, as the different results of ultrasound and pressure measurements on alike animal groups by Hartley and Wang suggest. Deviations to the findings can also be caused by the differences in age of the animals, because different animal strains were used, or because the animals were positioned vertically in the MR system. The PWVs measured in vivo in this study highly deviate from values found in an MR study that analyzed the part of the pulse wave that contains wave reflections [38].

Wang et al. found no significant differences in PWV values between ApoE^(-/-) ^and C57Bl/6J mice that were younger than 13 months [24]. In this study, a significant difference was observed at eight months of age already. In the current work, ApoE^(-/-) ^mice received a different, a western type diet, which accelerates aortic wall remodelling and increases elastic destruction and thus the PWV [41].

To make a general remark about the field of application of the TT method, it has to be considered that the TT method delivers a regional PWV value, which is averaged over the propagation pathway in between the measurement locations. In early stages of atherosclerosis diminutive vascular lesions are scattered along the aorta, which affect the PWV locally. Therefore, interest of research is also on the local elastic properties of the murine aorta. Our workgroup successfully applied the QA method [17] to mice in another study [22] to determine local PWVs. The QA method uses the volume flow (Q) to cross-sectional area (A) relation during the early systole to calculate the PWV. The QA method is unrivalled in local estimation of the PWV. But since it employs cross-sectional vessel areas, it comprises an additional source of error. The TT method is more robust and requires less user interpretation because it operates without the determination of cross-sectional areas [42].

## Conclusions

This study demonstrates the feasibility of the non-invasive determination of the pulse wave velocity by the transit time method and high-field CMR. The measured PWVs are accurate and reproducible in comparison to reference pressure measurements on a vessel phantom and sufficiently precise to distinguish between animal groups. Therefore, the measured PWV can be used as a marker to classify the state of arterial dysfunction in living mice. Due to the short in vivo acquisition times of 15 to 20 minutes, the presented MR method is applicable to enhance studies of morphologic parameters characterizing the murine arterial system [21,43] by adding the PWV as a functional parameter to form a comprehensive set of parameters.

## Competing interests

The authors declare that they have no competing interests.

## Authors' contributions

MP designed and manufactured the vessel phantom, designed and programmed the MR sequence and data analysis software, performed the phantom and in vivo measurements and the data evaluation, and drafted the manuscript. VH participated in the design and development of the MR sequence and data analysis software, performed the in vivo measurements, and participated in the phantom design. GK participated in the design of the study, helped coordinating it, and performed animal handling and supervision before and during in vivo experiments. WRB conceived of the study, participated in its design and coordination, and helped to draft the manuscript. ER designed and manufactured the heart triggering and breath-gating unit, participated in the manufacturing of the vessel phantom, and coordinated the study. PMJ participated in the conception of the study, provided the MR system and custom-made MR hardware, and critically revised the manuscript for important intellectual content. All authors read and approved the final manuscript.
